# Complete genome sequence of a bacterial strain, *Kurthia intestinigallinarum*

**DOI:** 10.1128/mra.00008-25

**Published:** 2025-04-28

**Authors:** Rikuto Kamiura, Shumpei Sato, Shuxian Wang, Yui Takanashi, Reo Nishiwaki, Atsushi Shibai, Chikara Furusawa

**Affiliations:** 1 Center for Biosystems Dynamics Research, RIKEN 536444 , Suita, Japan; 2 Faculty Division of Natural Science, Nara Women’s University, Kita-Uoya Nishi-machi, Japan; 3 Department of Biosciences and Informatics, Keio University, Yokohama, Japan; 4 Faculty of Agriculture, Kyoto University 12914, Kyoto, Japan; 5 Universal Biology Institute, Graduate School of Science, The University of Tokyo 13143, Tokyo, Japan; The University of Arizona, Tucson, Arizona, USA

**Keywords:** genome analysis, Kurthia, bacteria, complete genome

## Abstract

*Kurthia intestinigallinarum* was isolated from the oral cavity of a deer. In this article, we report the complete genome sequence of *K. intestinigallinarum*. This is the first complete genome report for this species.

## ANNOUNCEMENT

*Kurthia* is an aerobic genus of gram-positive bacteria isolated from soil, meat, and feces (1). Some species are associated with meat spoilage (1) and are suspected to have pathogenic potential (2); however, their physiological roles remain largely unexplored. In this study, we report the complete genome of *K. intestinigallinarum*. Its draft genome was previously reported (DDBJ/GenBank assembly: GCA_019114605.1), and the bacteria was isolated from the chicken gut.

We collected bacterial samples from the oral cavity of a Japanese deer (*Cervus nippon*). Using a sterile inoculation loop, we swabbed the deer’s oral vestibule and streaked the sample onto a TGY (Tryptone Glucose Yeast extract) agar plate. The plate was then incubated at room temperature (25 ˚C) for 2 days, and once colonies had developed, it was stored at 4 ˚C for preservation.

The stored cells were inoculated and cultured in 5 mL of TGY medium (0.5% tryptone, 0.1% glucose, 0.5% yeast extract, and 0.1% KH_2_PO_4_) at 30 ˚C overnight; 1 mL of the culture was pelleted by centrifugation, and genomic DNA was extracted using the Wizard Genomic DNA Purification Kit (Promega). For long-read sequencing, a library was prepared with a Native Barcoding Kit (SQK-NBD114.24, Oxford Nanopore Technologies) and sequenced on a MinION Mk1B with a Flow Cell (R10.4.1, FLO-MIN114). Base-calling and adapter trimming were performed using Dorado software (v0.7.2, Oxford Nanopore Technologies) in a super-accurate (SUP) mode. For short-read sequencing, we prepared a sample library for Illumina pair-end sequencing (2 × 300 bp) with Nextera XT kit (Illumina). An Illumina MiSeq sequenced the sample using MiSeq Reagent Kit v3 with 600 cycles. The MinION sequencing generated 145,353 reads with an average length of 7,515.6 bp and N50 of 16,855 bp. The MiSeq sequencing generated 1,688,664 paired short reads with an average length of 247 bp. We performed a hybrid *de novo* assembly of the short and long reads using Unicycler (ver. 0.5.1) (3) with SPAdes (ver. 4.0.0) (4) and Racon (ver. 1.3.3) (5). The genome coverage was 390×. Default parameters were used except where otherwise noted, including the following sentences.

Annotation was performed using the DDBJ Fast Annotation and Submission Tool (DFAST) (6) and Type (Strain) Genome Server (TYGS) (7, 8). The assembled GC poor (39.0%) genome had a total length of 3,855,051 bp and included 3,774 coding regions across seven sequences. The circularities were confirmed by Unicycler. DFAST identified the strain as *K. intestinigallinarum* (Genome Taxonomy Database Accession GCA_019114605.1), with an ANI of 98.79%. TYGS analysis confirmed this identification, with a digital DNA-DNA hybridization (dDDH, d4) value of 89.8%.

According to the previous criterion (9), the DNAs from the isolated strain can be divided into one chromosome, three megaplasmids, and three plasmids (Table 1). BUSCO (10) determined that just the chromosome contains core genes with the completeness of 100% (database: bacteria_obd10).

**TABLE 1:** Taxis of assembled DNA sequences

Sequence	Class	Length [bp]	Coding region	DDBJ/GenBank accession
Chromosome 1	Chromosome	2,429,201	2,458	AP038797
Plasmid 1	Megaplasmid	501,978	538	AP038798
Plasmid 2	Megaplasmid	469,216	430	AP038799
Plasmid 3	Megaplasmid	370,956	399	AP038800
Plasmid 4	Plasmid	42,054	58	AP038801
Plasmid 5	Plasmid	35,557	35	AP038802
Plasmid 6	Plasmid	3,659	3	AP038803

*K. intestinigallinarum* is useful as a natural case of multi-plasmid holder bacteria. Harboring some plasmids can be important to adapt hosts to some environments (11). Additionally, its presence in oral and intestinal environments makes it relevant for studying host-microbe interactions, with its genome sequence aiding in physiological function analysis.

## Data Availability

The complete genome sequence of *Kurthia intestinigallinarum* was deposited as a set (a chromosome, three megaplasmids and three plasmids) in DDBJ/GenBank under accession numbers AP038797 to AP038803. The fastq files of the raw reads were deposited in the DDBJ Sequence Read Archive (DRA)/NCBI SRA under accession numbers DRR618345 and DRR618346.

## References

[1] Stackebrandt E, Keddie RM, Jones D. 2006. The genus *Kurthia*, p 519–529. In Dworkin M, Falkow S, Rosenberg E, Schleifer KH, Stackebrandt E (ed), The prokaryotes: volume 4: bacteria: firmicutes, cyanobacteria. Springer US.

[2] Lozica L, Maurić Maljković M, Mazić M, Gottstein Ž. 2022. Kurthia gibsonii, a novel opportunistic pathogen in poultry. Avian Pathol 51:26–33. doi:10.1080/03079457.2021.1993132 34662527

[3] Wick RR, Judd LM, Gorrie CL, Holt KE. 2017. Unicycler: resolving bacterial genome assemblies from short and long sequencing reads. PLoS Comput Biol 13:e1005595. doi:10.1371/journal.pcbi.1005595 28594827 PMC5481147

[4] Prjibelski A, Antipov D, Meleshko D, Lapidus A, Korobeynikov A. 2020. Using SPAdes de novo assembler. Curr Protoc Bioinformatics 70:e102. doi:10.1002/cpbi.102 32559359

[5] Vaser R, Sović I, Nagarajan N, Šikić M. 2017. Fast and accurate de novo genome assembly from long uncorrected reads. Genome Res 27:737–746. doi:10.1101/gr.214270.116 28100585 PMC5411768

[6] Tanizawa Y, Fujisawa T, Nakamura Y. 2018. DFAST: a flexible prokaryotic genome annotation pipeline for faster genome publication. Bioinformatics 34:1037–1039. doi:10.1093/bioinformatics/btx713 29106469 PMC5860143

[7] Meier-Kolthoff JP, Göker M. 2019. TYGS is an automated high-throughput platform for state-of-the-art genome-based taxonomy. Nat Commun 10:2182. doi:10.1038/s41467-019-10210-3 31097708 PMC6522516

[8] Meier-Kolthoff JP, Carbasse JS, Peinado-Olarte RL, Göker M. 2022. TYGS and LPSN: a database tandem for fast and reliable genome-based classification and nomenclature of prokaryotes. Nucleic Acids Res 50:D801–D807. doi:10.1093/nar/gkab902 34634793 PMC8728197

[9] diCenzo GC, Finan TM. 2017. The divided bacterial genome: structure, function, and evolution. Microbiol Mol Biol Rev 81:e00019-17. doi:10.1128/MMBR.00019-17 28794225 PMC5584315

[10] Manni M, Berkeley MR, Seppey M, Simão FA, Zdobnov EM. 2021. BUSCO update: novel and streamlined workflows along with broader and deeper phylogenetic coverage for scoring of eukaryotic, prokaryotic, and viral genomes. Mol Biol Evol 38:4647–4654. doi:10.1093/molbev/msab199 34320186 PMC8476166

[11] Wiedenbeck J, Cohan FM. 2011. Origins of bacterial diversity through horizontal genetic transfer and adaptation to new ecological niches. FEMS Microbiol Rev 35:957–976. doi:10.1111/j.1574-6976.2011.00292.x 21711367

